# Incidence of non-cardia gastric cancer among commercially-insured individuals aged 18–64 with chronic atrophic gastritis

**DOI:** 10.1371/journal.pone.0315833

**Published:** 2025-06-23

**Authors:** Robert J. Huang, Vidhya Balasubramanian, Miranda V. Shum, Hanlee P. Ji, Joo Ha Hwang

**Affiliations:** 1 Division of Gastroenterology and Hepatology, Stanford University School of Medicine, Stanford, California, United States of America; 2 Division of Oncology, Stanford University School of Medicine, Stanford, California, United States of America; Al-Azhar University, EGYPT

## Abstract

**Background:**

Chronic atrophic gastritis (**CAG**) is a precancerous condition of the gastric mucosa which predisposes to non-cardia gastric cancer (**NCGC**). The risk for NCGC following diagnosis with CAG has not been described robustly in the United States.

**Methods:**

We used a commercial claims database (Marketscan, Merative LP) covering over 150 million privately-insured Americans aged 18–64 to create a cohort of individuals diagnosed with CAG. We then followed these individuals for the development of NCGC or to the time of their last clinical encounter. Demographic and clinical characteristics were captured through administrative coding schema, and linked to metropolitan statistical area measures of socioeconomic status. Individual race and ethnicity were not available for this analysis.

**Findings:**

We analyzed data on 107,835 individuals and recorded 355,591 person-years (p-y) of follow-up. The crude overall incidence of NCGC was 98 per 100,000 p-y. In the fully-adjusted multivariable proportional hazards model, age ≥ 50 (HR 2.20, 95% CI 1.44–3.36), anemia (HR 5.09, 95% CI 3.46–7.50), former or current smoking (HR 1.42, 95% CI 1.11–1.81) and family history (HR 1.44, 95% CI 1.05–1.99) were individual-level factors associated with increased risk.

**Conclusions:**

We present one of the first estimates of NCGC risk following CAG diagnosis in an American population, and highlight risk factors for cancer progression. These data may help to guide future risk prevention strategies, such as endoscopic surveillance, in the United States.

## Introduction

Chronic atrophic gastritis (**CAG**) is an important precursor to non-cardia gastric cancer (**NCGC**). CAG is defined by the loss of normal gastric glandular structures and replacement by either connective tissue or non-native epithelium (metaplastic atrophy) in response to chronic inflammation and injury [1]. CAG represents an important first step in a carcinogenic pathway, termed Correa’s cascade, which ultimately leads to the development of adenocarcinoma through progressive histologic insult and cellular injury [2]. Worldwide, infection by *Helicobacter pylori* (***H. pylori***) appears to be the dominant cause of CAG; however, in regions of the world with lower *H. pylori* prevalence, autoimmune gastritis may also be a significant risk factor [1]. The worldwide prevalence of CAG varies, with estimates of ~5% in low-NCGC-incidence populations to as high as ~50% in high-incidence populations, and a pooled global prevalence of ~25% [3,4]. The CAG prevalence in the United States (**US**) has been estimated at ~10–15%, with variability based on race, ethnicity, and immigrant status [1].

The incidence of progression from CAG to NCGC has been a topic of research interest, as defining more precise estimates for progression can help to guide targeted prevention efforts such as endoscopic screening. Two large European national registry studies have provided estimates of this risk. One study from the Netherlands followed some 22,000 patients with CAG and reported 1-, 5-, and 10-year risk of NCGC at 0.3%, 0.6%, and 0.8%, respectively [5]. A second population-based cohort study in Sweden identified 14,000 patients with CAG at baseline, and observed a crude incidence of NCGC at 90 per 100,000 person-years (**p-y**) [6]. There have also been smaller cohort studies from European populations [7–9]; however, their smaller sizes (n ≤ 300) make challenging the detection of cancer events. There have been two US-based cohort studies focused on metaplastic CAG (termed gastric intestinal metaplasia, **GIM**) [10,11], a more advanced lesion compared to CAG with higher cancer risk. However, it is unclear if these risk estimates are applicable to CAG; moreover, in these two studies the number of cancer events was < 20 [10,11]. Despite the interest in this topic, there have been no population-based cohort studies on the rate of progression from CAG to NCGC conducted in the US. Moreover, European-based data may only have limited generalizability to the US population, as a recent meta-analysis demonstrated regional differences in the risk for CAG progression toward cancer [12].

The Merative™ Marketscan® Research Databases Commercial Claims and Encounters database represents one of the largest repositories of commercial-payer data in the US. The database contains deidentified outpatient, inpatient, and prescription claims data from US-based employers who provide health plans for their employees and their families. Notably the database only contains clinical data on individuals <65 years of age, as Medicare-linked plans are not included. Also importantly, MarketScan does not report race or ethnicity data [13]. MarketScan does provide geospatial data on residence of covered individuals, including metropolitan statistical area (**MSA**).

In this population-based cohort study, we utilized the Commercial Claims and Encounters database to estimate the risk of transition from CAG to NCGC in a privately-insured population of individuals <65 years of age. We performed both cohort creation and outcome ascertainment using administrative coding, with a multi-step process to minimize misclassification.

## Materials and methods

### Research ethics and data availability

The study uses only observational data from deidentified individuals and geographic-level estimates of socioeconomic status derived from the American Community Survey (**ACS**). This study was determined by the Stanford Institutional Review Board to not involve human subjects (determination number 59085) as the data were professionally and irrevocably deidentified prior to access by the researchers. The MarketScan database was accessed through the Stanford Center for Population Health Science [14], which reviewed and approved the submission of this manuscript. Data from the ACS was obtained from the US Census Bureau [15], and is available for public access. The funders of this study had no role in the collection or analysis of the data, had no role in the writing of the manuscript, and did not influence the decision to submit the manuscript for publication.

### Data sources

The MarketScan Commercial Claims and Encounters database (years 2006–2022) contain deidentified data on over 150 million employees and dependents in the US covered through privately insured fee-for-service, point-of-service, or capitated health plans. These data sets integrate many types of data including outpatient clinic records, inpatient admission and discharge data, procedural claims, and pharmaceutical claims. Notably, the Commercial Claims and Encounters database only covers individuals <65 years of age, as individuals transition to Medicare beginning at age 65 [16,17]. Also notably, MarketScan data contains no information on an individual’s race or ethnicity.

The ACS is the largest source of small area statistics for demographic characteristics across the US and is based on sampling of ~3.5 million households annually. MSAs are geographic entities based on a county or group of counties with at least one urbanized area with a population of at least 50,000, and with adjacent counties with economic ties to the central area. In year 2020, it has been estimated that ~86% of the US population resides within one of the 387 MSAs [18]. We used 2019 ACS data to ascertain economic characteristics of MSAs which were then linked to individual-level residence data form MarketScan.

### Inclusion and exclusion criteria

The overall study design is depicted in graphical format in Fig S1. Eligible individuals were those that 1) were recorded to have a diagnosis of CAG based on international classification of disease 9^th^ (**ICD-9**) and 10^th^ (**ICD-10**) revision codes (a comprehensive list of all ICD-9 and ICD-10 codes in this study are presented in Table S1), and 2) were at least 18 years of age at time of CAG diagnosis. From these individuals we excluded those with <180 days of follow-up (defined as no subsequent clinical encounter after 180 days), individuals with a prior NCGC diagnosis (previous to CAG diagnosis), and individuals with NCGC diagnosis within 180 days of CAG diagnosis (we defined these as ‘prevalent’ cancer diagnoses, and removed them from analysis similar to prior studies) [6,10]. To minimize misclassification, we also required a second diagnosis of CAG from either outpatient or inpatient records on a separate day from the initial diagnosis. We also removed individuals missing MSA data from analysis.

### Cohort variable collection

We captured demographic, clinical, pharmaceutical, and geographic variables from the cohort. We plotted the age distribution of the cohort (Fig S2), and found both the median to be 48 years and mean to be 50 years. As 50 is the age at which certain countries (such as Japan) [19] begin screening for NCGC, we adopted this as a cutoff for dichotomization of the age variable. Clinical variables captured included several established risk factors for NCGC: prior *H. pylori* infection [20], anemia [21], a family history of digestive neoplasm, former or current smoking, and obesity [22–24]. These variables were captured through ICD-9 and ICD-10 codes (Table S1), using previously validated schema [21, 25–29]. While some patients with CAG will likely also have GIM, we could not separately capture GIM as a diagnosis in this study, as the first ICD-10 code specific for GIM was only promulgated in late 2021 [30]. Variables were treated as non-time-varying for the purposes of analysis. Pharmaceutical claims were captured through a combination of national drug codes and therapeutic class variable found in MarketScan. For medications, we captured dispensation records on four commonly prescribed medicines which have demonstrated association (either positive or negative) with NCGC risk [22–24]: aspirin, non-steroidal anti-inflammatory drugs (**NSAIDs**), proton-pump inhibitors (**PPIs**), and selective histamine type 2 receptor antagonists (**H2RA**). For the aspirin variable, we were not able to capture any information on over-the-counter aspirin purchases and use. We also captured data on dispensation of antibiotics for *H. pylori* eradication therapy. Individuals were classified as having detected *H. pylori* infection if either 1) they carried an ICD-9 or ICD-10 code of *H. pylori* infection, or 2) they received a dispensed prescription for *H. pylori* eradication therapy. Patients were otherwise classified as having no prior *H. pylori* detection. From the MSA-level variables, we captured median household income (in 2019 dollars) as a marker of socioeconomic status.

### Outcome ascertainment and statistical analysis

The primary outcome measure was development of NCGC greater than 180 days after entry (defined as the first date of CAG diagnosis). NCGCs diagnosed within 180 days of enrollment were considered prevalent and excluded (Fig S1) [6,10]. To minimize misclassification, we required NCGC diagnoses to occur in two separate clinical encounters on two separate dates, with the date of diagnosis defined as the first of these encounters. If no NCGC occurred, the date of censoring was the last available encounter (either inpatient or outpatient) date. Our primary analysis included codes which could potentially overlap with cardia tumors (listed in Table S1). As a sensitivity analysis, we removed these codes (151.9, C16.8, C16.9) from the outcome definition for analytic purposes.

We described the study cohort with frequencies for categorical variables, and mean and standard deviation for continuous variables. The person-years at risk, incident NCGC cases, and incidence rates per 100,000 p-y were calculated within each category. Cumulative hazards plots for each risk factor were generated and compared between categories by the log-rank test. We performed both univariate and multivariate Cox proportional hazards regression to estimate the hazard ratios (**HR**) and 95% confidence intervals (**CI**) for NCGC for covariates. Schoenfeld residuals were computed to test the proportional hazards assumption. In multivariable regression, we assessed for statistical interaction between variables of *a priori* interest, as well as variables which demonstrated significant univariable association. All analyses are performed using RStudio Version 1.2.5033. Survival analyses used the “survival” library.

## Results

### Cohort discovery

**Fig 1:**
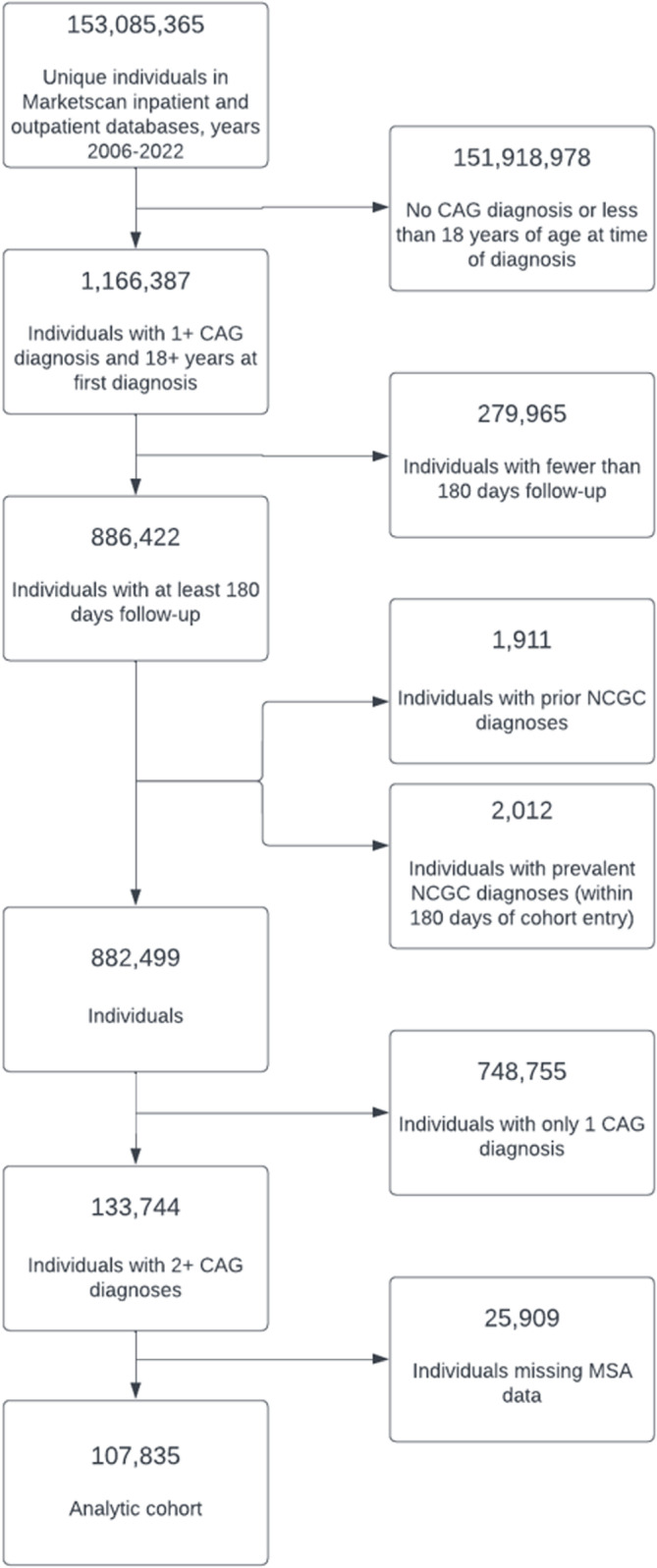
Flow diagram demonstrate numbers of individuals at each stage of the study. The number included, and number excluded at each step (along with reason for exclusion) are presented. The number completing follow-up and analyzed are shown. CAG, chronic atrophic gastritis; MSA, metropolitan statistical area; NCGC, non-cardia gastric cancer.

A flow diagram demonstrating numbers of eligible and excluded individuals at each step of cohort refinement is depicted in Fig 1. From 153 million unique enrollees in the Commercial Claims and Encounters database, 1,166,387 carried at least one diagnosis of CAG. Of these, 279,965 did not have at least 180 days of follow-up data. Another 1,1911 patients had a personal history of NCGC recorded, and 2,012 patients had a diagnosis of NCGC made within 180 days and were excluded. From these 882,499 patients, 748,755 did not have a second CAG diagnosis and 25,909 were missing MSA information. Our final analytic cohort comprised of 107,835 individuals.

### Cohort characteristics and crude incidence

Cohort characteristics and incidence by characteristic are depicted in Table 1. The cohort constituted 107,835 individuals and 355,591 p-y of observation. We observed 348 incident NCGC cases, and overall incidence was 98 per 100,000 p-y. The cohort was predominantly female (65%), with roughly equal proportion above and below 50 years of age. Both males and those aged 50–64 had higher NCGC incidence. About 54% of the cohort had detected *H. pylori* infection, and the incidence of NCGC was actually lower in this group (87 per 100,000 p-y) compared to the overall cohort. Individuals with anemia, smoking history, and family history of digestive neoplasm had higher incidence of NCGC. Regarding medication usage, PPI (44%), H2RA (15%), and NSAID (46%) use was common. Recorded aspirin use was very low (1%), but this may be due to over-the-counter purchases not being recorded in the pharmaceutical claims data. The median household income was $66,200 (2019 US dollars).

**Table 1:** Cohort characteristics and incidence rates.

Characteristic (%)	Number	P-y of observation	NCGC Cases (%)	Incidence (per 100,000 p-y)
**Overall**	107,835 (100.0)	355,591	348 (100.0)	97.9
**Demographic**				
Female	69,548 (64.5)	232,830	184 (52.9)	79.0
Male	38,287 (35.5)	122,761	164 (47.1)	133.6
Age < 50	50,508 (46.8)	168,484	94 (27.0)	55.8
Age 50–64	57,327 (53.2)	187,107	254 (73.0)	135.8
**Medical history**				
*H. pylori* detected*	57,953 (53.7)	209,495	183 (52.6)	87.4
Anemia	48,267 (44.8)	165,275	263 (75.6)	159.1
Smoking (current/prior)	19,934 (18.5)	670,780	89 (25.6)	132.7
Obesity	33,461 (31.0)	120,153	97 (27.9)	80.7
Family history digestive neoplasm	8,528 (7.9)	32,937	43 (12.4)	130.6
**Medication use**				
PPI user	47,069 (43.6)	173,662	146 (42.0)	84.1
H2RA user	15,747 (14.6)	62,067	46 (13.2)	74.1
Aspirin user**	1,070 (1.0)	4,545	6 (1.7)	132.0
NSAID user	50,013 (46.4)	190,069	140 (40.2)	73.7

Characteristics of cohort and incidence rates. *H. pylori detection defined as either presence of diagnosis code or dispensation of eradication therapy. **Does not include non-prescription (over-the-counter) aspirin purchases. H2RA, H2 receptor antagonist; MSA, metropolitan statistical area; NCGC, non-cardia gastric cancer; NSAID, non-steroidal anti-inflammatory drug; PPI, proton pump inhibitor; p-y, person-year.

### Cumulative hazard

**Fig 2:**
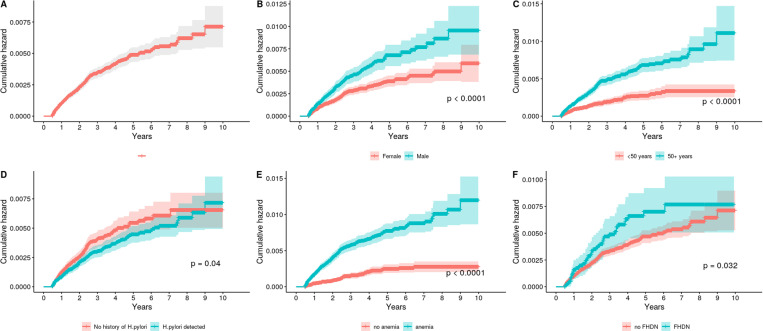
Cumulative hazard plots for non-cardia gastric cancer for the entire cohort (panel A), by sex (B), by age (C), by *H. pylori* detection status (D), by history of anemia (E), and by family history of digestive neoplasm (FHDN, F). Tests of significance (log-rank p values) are found in the lower right corner of each panel. Corresponding table of numbers at risk are found in Table S2.

Cumulative hazard plots by individual-level characteristics are depicted in Fig 2, and associated at-risk tables are shown in Table S2. Males (log-rank p < 0.0001), individuals ≥50 years (p < 0.0001), individuals with anemia (p < 0.001), and those with a family history of digestive neoplasm (p = 0.032) had a higher risk for developing NCGC. Interestingly, history of detected *H. pylori* was not associated or even slightly inversely-associated with NCGC risk (log-rank p = 0.04).

### Cox regression and sensitivity analysis

Univariable and multivariable proportional hazards regression are depicted in Table 2. In univariable regression, male sex (p < 0.001), age 50−64 (p < 0.001), anemia (p < 0.001), smoking (p = 0.001), and a family history (p = 0.03) were significantly associated with increased NCGC risk. Previously detected *H. pylori* infection (HR 0.80, 95% CI 0.65–0.99) and obesity (HR 0.77, 95% CI 0.61–0.98) were inversely associated. NSAID use was inversely associated (HR 0.62, 95% CI 0.48–0.81); no other medication use demonstrated association. At the MSA level, increasing median household income demonstrated *positive* association with risk (HR 1.09, 95% CI 1.01–1.17; per 10,000 US dollars).

**Table 2:** Cox Proportional hazards univariable and multivariable estimates.

Characteristic	Univariable		Multivariable	
	HR (95% CI)	p-value	HR (95% CI)	p-value
**Demographic or Clinical**				
Male sex	1.68 (1.36, 2.07)	<0.001	1.86 (1.01, 3.44)	0.05
Age 50–64	2.40 (1.89, 3.04)	<0.001	2.20 (1.44, 3.36)	<0.001
*H. pylori* detected*	0.80 (0.65, 0.99)	0.04	0.94 (0.60, 1.46)	0.8
Anemia	3.61 (2.82, 4.60)	<0.001	5.09 (3.46, 7.50)	<0.001
Smoking	1.48 (1.16, 1.88)	0.001	1.42 (1.11, 1.81)	0.005
Obesity	0.77 (0.61, 0.98)	0.03	0.75 (0.59, 0.95)	0.02
Family history digestive neoplasm	1.42 (1.03, 1.95)	0.03	1.45 (1.05, 1.99)	0.03
**Medication use**				
PPI	0.90 (0.69, 1.17)	0.4	---	
H2RA	0.82 (0.59, 1.13)	0.2	---	
Aspirin	1.57 (0.70, 3.52)	0.3	---	
NSAID	0.62 (0.48, 0.81)	<0.001	---	
**MSA Characteristic**				
Median Household Income (per 10k, continuous)	1.09 (1.01, 1.17)	0.034	1.07 (0.99, 1.16)	0.07
**Interaction terms**				
Male | Anemia	---		0.55 (0.33, 0.91)	0.02
Male | *H. pylori*	---		1.18 (0.77, 1.81)	0.5
50-64 years | *H. pylori*	---		0.74 (0.45, 1.20)	0.2
Male | 50–64 years	---		1.46 (0.89, 2.39)	0.1

Univariable and multivariable regression. *H. pylori detection defined as either presence of diagnosis code or dispensation of eradication therapy. H2RA, H2 receptor antagonist; HR, hazard ratio; MSA, metropolitan statistical area; NCGC, non-cardia gastric cancer; NSAID, non-steroidal anti-inflammatory drug; PPI, proton pump inhibitor.

In the multivariable model, male sex (HR 1.86, 95% CI 1.01–3.44), age 50−64 (HR 2.20, 95% CI 1.44–3.36), anemia (HR 5.09, 95% CI 3.46–7.50), smoking (HR 1.42, 95% CI 1.11–1.81), and family history (HR 1.45, 95% CI 1.05–1.99) continued to demonstrate significant association with NCGC risk. MSA household income did not demonstrate association. There existed a significant interaction between male sex and anemia (HR 0.55, 95% CI 0.33–0.91 for interaction term).

In a sensitivity analysis, we excluded potential overlap codes of 151.9 (ICD-9), and C16.8/C16.9 (ICD-10). This sensitivity analysis is presented in Table S3. In the univariable analysis male sex, older age, anemia, and smoking remained significantly associated with NCGC risk. Obesity and NSAID use continued to demonstrate inverse association. MSA household income demonstrated no association. In the multivariable model, older age, anemia, smoking, and obesity remained significantly associated.

## Discussion

In this population-based cohort study, we report on the rate of progression to NCGC in a nationwide cohort of 108,000 privately-insured US persons aged 18–64 diagnosed with CAG. Over 355,000 p-y of observation, the aggregate crude incidence of NCGC was 98 per 100,000 p-y. Older individuals, those with anemia, smokers, and those with a family history of digestive neoplasm were at increased risk for disease progression.

In the context of existing literature, our study adds important data on cancer progression. We believe this to be the first population-based cohort study of CAG progression in a US population. It is also, by a significant margin, the largest cohort study of CAG progression in any region of the world; as comparison, a recent meta-analysis of worldwide literature on progression rates included data from 21 studies and ~57,000 individuals with CAG [12]. The risk estimates provided in this US-based study are comparable to the progression rates reported in low-incidence regions reported in this meta-analysis (97 per 100,000), and lower than the rate reported in medium/high-incidence regions (247 per 100,000) [12]. It is important to note however that our population was restricted to individuals <65 years of age, and age is a risk factor for progression (both in our cohort and prior studies). It seems probable that had we access to a data set inclusive of older individuals residing in the US, the aggregate risk for NCGC would be higher than our estimate.

An additional advantage of our study is the ability to isolate risk factors for cancer progression. There exists sparse data on risk factors for cancer progression once CAG has developed. Most of this existing data is derived from GIM, a subset of CAG which is believed to be of higher risk. Recent American [1,31,32] and European [33,34] guidelines have suggested family history of NCGC, GIM extent and histologic severity, and *H. pylori* persistence to be features of higher-risk GIM lesions. Our study defines progression risk factors for the preceding step of CAG. These risk factors include age ≥ 50, anemia, current or prior smoking, and a family history of digestive neoplasm. Currently, there are no guidelines in the US that specify which patients with CAG require endoscopic surveillance. The cancer progression estimates presented in this study, stratified by risk factor status, may be compared with progression rates for higher lesions (such as GIM) [12,31], and be used to triage CAG patients for targeted risk attenuation strategies such as endoscopic surveillance.

Notably, previously-detected *H. pylori* diagnosis (either through coding or eradication therapy prescription) did not demonstrate an association with NCGC risk. We posit several hypotheses for this observation. As *H. pylori* is an established carcinogen, clinical guidelines currently recommend eradication of the infection if detected for any reason [35]. The *H. pylori*-detected group therefore mostly represents individuals with historical infection; by contrast, the non-detected group may contain individuals with occult infection. Prior infection could also be under-captured from coding schema, as CAG is known to persist for years or even decades after *H. pylori* eradication [36]. As our study window capture data only up to 2006, individuals with infection eradicated before this time would not be captured. An additional possibility is that a subset of the *H. pylori*-negative CAG patients may have autoimmune gastritis. This is a body-predominant form of inflammation driven by auto-antibody formation to parietal cells and intrinsic factor, with significant risk for both adenocarcinoma and neuroendocrine tumors [1]. One prominent feature of autoimmune gastritis is the development of anemia, either through vitamin B12 deficiency (pernicious anemia) or through inadequate iron absorption. We found anemia to be a highly-significant predictor for NCGC whose association actually strengthened in the multivariable analysis. Unfortunately, there is no reliable mechanism to differentiate between *H. pylori*-induced vs autoimmune CAG using administrative codes.

In our study, we found obesity to be inversely associated with NCGC progression risk. We are not aware of prior studies to have demonstrated this association in the context of CAG, but a prior meta-analysis of published cohort studies in general populations (not with known CAG) found no association between excess body weight and NCGC [37]. While we did find NSAID use to be inversely associated with cancer risk in the univariable analysis, there were insufficient data to evaluate NSAID use in multivariable analyses. Cohort studies on the effect of NSAID use in general populations have yielded conflicting results, with one study (based on the Multethnic Cohort) showing an inverse association for aspirin but not for non-aspirin NSAID use [38], and another study (based on the NIH-AARP Diet and Health Study) showing an inverse association for both aspirin and NSAID use [39]. The very low prevalence of aspirin use in our cohort, likely reflective of over-the-counter aspirin administration, limited our ability to analyze aspirin as a protective factor.

Our study has notable limitations related to study design. Ascertainment of exposure and outcomes were based on administrative codes, with possible misclassification. We attempted to utilize a strategy requiring multiple codes entered on different dates to enhance validity; nevertheless, coding-based misclassification remains a concern. The cohort was constituted of privately-insured Americans <65 years of age. Risk estimates incorporating uninsured or publicly-insured individuals, including those 65 and older on Medicare, are needed to design policies focused on early detection and screening. No histologic information on CAG is available based on administrative coding. For instance, we could not determine whether CAG was extensive (involving antrum and body) or limited, or whether CAG was histologically mild or severe. A scoring system which combines both histologic severity and anatomic extent, termed the operative link on gastric atrophy, has demonstrated promise as a risk stratification tool [40]. Another limitation of the study is the inability to capture synchronous GIM. Surprisingly until 2021, there existed no distinct ICD-10 code for GIM [41].

In summary, we present a large cohort of US-based, privately-insured individuals aged 18–64 diagnosed with CAG and followed to a cancer endpoint. We believe the data from this study may serve as a valuable resource to define and estimate risk, and target higher-risk groups for prevention efforts.

## Supporting information

S1 Table ICD-9 and ICD-10 codes. (PDF)

S2 Table Numbers at risk and incident cancers. (PDF)

S3 Table Cox regression sensitivity analysis (excluding 151.9, C16.8, C16.9). (PDF)

S1 Fig Patient timeline with cohort inclusion and endpoint definitions. (PDF)

S2 Fig Age distribution of the cohort. (PDF)

## Abbreviations

ACS: American Community Survey
CAG: Chronic atrophic gastritis
CI: Confidence interval
*H. pylori*: *Helicobacter pylori*
H2RA: Histamine type 2 receptor antagonists
HR: Hazard ratio
ICD-9: International Classification of Diseases, Ninth Revision
ICD-10: International Classification of Diseases, Tenth Revision
MSA: Metropolitan statistical area
NCGC: Non-cardia gastric cancer
NSAID: Non-steroidal anti-inflammatory drug
p-y: Person-years
PPI: Proton-pump inhibitor
US: United States.
