# Supplementary material for: Incidence of non-cardia gastric cancer among commercially-insured individuals aged 18–64 with chronic atrophic gastritis
Source: PLoS One. 2025 Jun 23;20(6):e0315833. doi: 10.1371/journal.pone.0315833 (PMC12185002; doi:10.1371/journal.pone.0315833)
Supplement: S1 Table — (PDF) [file pone.0315833.s001.pdf]

Table S1: ICD-9 and ICD-10 codes		
**Condition**	**ICD-9**	**ICD-10**
Chronic Atrophic Gastritis	535.10, 535.11	K29.4, K29.41
Non-cardia gastric cancer	151.1, 151.2, 151.3, 151.4, 151.5, 151.6, 151.8, 151.9	C16.1, C16.2, C16.3, C16.4, C16.5, C16.6, C16.8, C16.9
Non-cardia gastric cancer, excluding overlap codes	151.1, 151.2, 151.3, 151.4, 151.5, 151.6, 151.8	C16.1, C16.2, C16.3, C16.4, C16.5, C16.6
Heliobacter pylori infection	041.86	B96.81
Anemia	280–281, 283–285	D50-D64
Smoking	305.1, V15.82, 649.0X, 989.84	F17.20, F17.200, F17.201, F17.203, F17.208, F17.209, F17.21, F17.210, F17.211, F17.213, F17.218, F17.219, F17.29, F17.290, F17.291, F17.293, F17.298, F17.299, Z72.0
Obese	278, V85	E66.01, E66.09, E66.1, E66.2, E66.8, E66.9, Z68.30, Z68.31, Z68.32, Z68.33, Z68.34, Z68.35, Z68.36, Z68.37, Z68.38, Z68.39, Z68.41, Z68.42, Z68.43, Z68.44, Z68.45
Family history of digestive neoplasm	V16.0	Z80.0

List of international classification of disease 9th (ICD-9) and 10th (ICD-10) revision codes captured in this study. References for choice of coding schema are in main text.
