# Supplementary material for: Incidence of non-cardia gastric cancer among commercially-insured individuals aged 18–64 with chronic atrophic gastritis
Source: PLoS One. 2025 Jun 23;20(6):e0315833. doi: 10.1371/journal.pone.0315833 (PMC12185002; doi:10.1371/journal.pone.0315833)
Supplement: S2 Table — (PDF) [file pone.0315833.s002.pdf]

Table S2: Numbers at Risk and Incident Cancers				
	**0 days**	**1,000 days**	**2,000 days**	**3,000 days**
**Overall**				
At Risk	107,835	55,366	18,028	3,525
Events	0	273	335	346
**Female**				
At Risk	69,548	36,307	12,099	2,409
Events	0	147	178	183
**Male**				
At Risk	38,287	19,059	5,929	1,116
Events	0	126	157	163
**Age <50 years**				
At Risk	50,508	25,929	8,990	1,979
Events	0	69	91	94
**Age 50-64 years**				
At Risk	57,327	29,437	9,038	1,546
Events	0	204	244	252
**No *H. pylori* history**				
At Risk	49,882	22,194	5,968	990
Events	0	138	162	165
***H. pylori* detected**				
At Risk	57,953	33,172	12,060	2,535
Events	0	135	173	181
**No Anemia**				
At Risk	59,568	29,319	9,173	1,783
Events	0	63	83	85
**Anemia**				
At Risk	48,267	26,047	8,855	1,742
Events	0	210	252	261
**No Smoking**				
At Risk	87,901	44,804	14,585	2,851
Events	0	208	249	257
**Smoking**				
At Risk	19,934	10,562	3,443	674
Events	0	65	86	89
**No Obesity**				
At Risk	743,74	36,353	11,174	2,130
Events	0	206	246	251
**Obesity**				
At Risk	33,461	19,013	6,854	1,395
Events	0	67	89	95
**No family history digestive neoplasm**				
At Risk	99,307	50,032	16,027	3,105
Events	0	240	293	303
**Family history digestive neoplasm**				
At Risk	8,528	5,334	2,001	420
Events	0	33	42	43

H. pylori detection includes those with either diagnosis codes or dispensation history.
