# Supplementary material for: Incidence of non-cardia gastric cancer among commercially-insured individuals aged 18–64 with chronic atrophic gastritis
Source: PLoS One. 2025 Jun 23;20(6):e0315833. doi: 10.1371/journal.pone.0315833 (PMC12185002; doi:10.1371/journal.pone.0315833)
Supplement: S3 Table — (PDF) [file pone.0315833.s003.pdf]

Table S3: Cox Regression Sensitivity Analysis (excluding 151.9, C16.8, C16.9)				
	**Univariable**		**Multivariable**	
**Characteristic**	HR (95% CI)	p-value	HR (95% CI)	p-value
**Demographic or Clinical**				
Male sex	1.92 (1.36, 2.71)	<0.001	2.32 (0.71, 7.54)	0.2
Age 50-64	3.07 (2.03, 4.64)	<0.001	4.03 (1.66, 9.75)	0.002
H. pylori detected*	1.09 (0.77, 1.56)	0.6	1.86 (0.75, 4.58)	0.2
Anemia	4.82 (3.11, 7.46)	<0.001	7.16 (3.41, 15.05)	<0.001
Smoking	1.66 (1.13, 2.45)	0.009	1.55 (1.05, 2.28)	0.03
Obesity	0.65 (0.44, 0.97)	0.04	0.63 (0.42, 0.94)	0.02
Family history digestive neoplasm	1.21 (0.69, 2.11)	0.5	1.24 (0.71, 2.16)	0.5
**Medication use**				
PPI	1.03 (0.67, 1.58)	0.9	---	
H2RA	0.89 (0.54, 1.48)	0.7	---	
Aspirin	2.02 (0.64, 6.39)	0.2	---	
NSAID	0.49 (0.32, 0.74)	<0.001	---	
**MSA Characteristic**				
Median Household Income (per 10K)	1.08 (0.96, 1.23)	0.2	1.08 (0.95, 1.22)	0.3
**Interaction terms**				
Male | Anemia	---		0.53 (0.21, 1.33)	0.2
Male | *H. pylori*	---		1.24 (0.60, 2.54)	0.6
50-64 years | *H. pylori*	---		0.46 (0.18, 1.19)	0.1
Male | 50-64 years	---		1.34 (0.57, 3.16)	0.5

Univariable and multivariable regression, excluding potential overlap codes. **H. pylori* detection defined as either presence of code or dispensation of eradication therapy. H2RA, H2 receptor antagonist; HR, hazard ratio; MSA, metropolitan statistical area; NCGC, non-cardia gastric cancer; NSAID, non-steroidal anti-inflammatory drug; PPI, proton pump inhibitor.
