# Supplementary material for: Incidence of non-cardia gastric cancer among commercially-insured individuals aged 18–64 with chronic atrophic gastritis
Source: PLoS One. 2025 Jun 23;20(6):e0315833. doi: 10.1371/journal.pone.0315833 (PMC12185002; doi:10.1371/journal.pone.0315833)
Supplement: S1 Fig — (PDF) [file pone.0315833.s004.pdf]

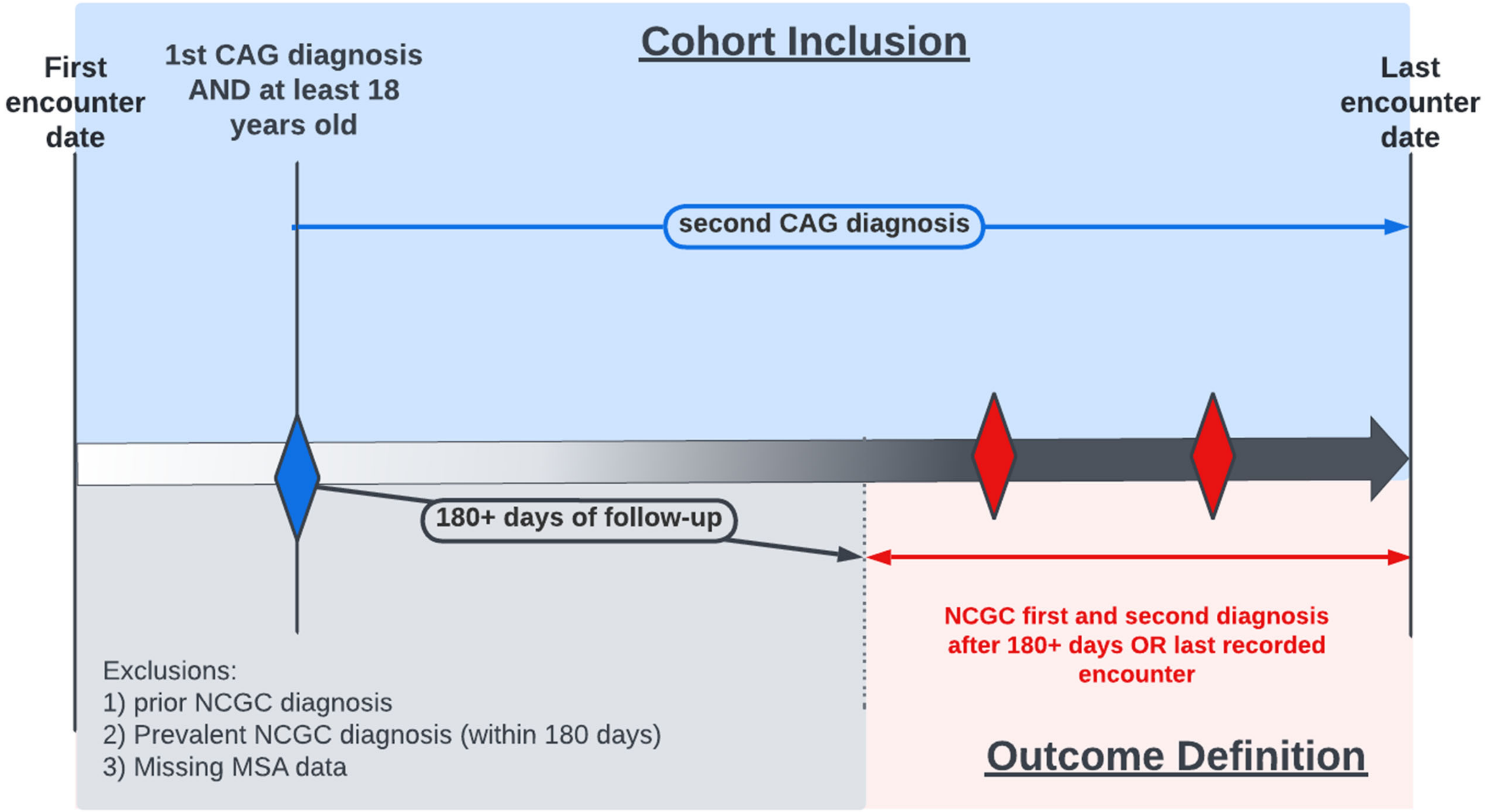

Figure S1. Patient timeline with cohort inclusion and endpoint definitions. Cohort inclusion requires two or more chronic atrophic gastritis (**CAG**) diagnoses. Outcome defined as two or more non-cardiac gastric cancer (**NCGC**) diagnoses, which occurs at least 180 days after first AG diagnosis. If no NCGC occurs, the last encounter data (inclusive of both outpatient and inpatient encounters) is used for censoring. Individuals with prior NCGC, prevalent NCGC diagnosis (within 180 days), and missing metropolitan statistical area (**MSA**) data were excluded.
