# Supplementary material for: Incidence of non-cardia gastric cancer among commercially-insured individuals aged 18–64 with chronic atrophic gastritis
Source: PLoS One. 2025 Jun 23;20(6):e0315833. doi: 10.1371/journal.pone.0315833 (PMC12185002; doi:10.1371/journal.pone.0315833)
Supplement: S2 Fig — (PDF) [file pone.0315833.s005.pdf]

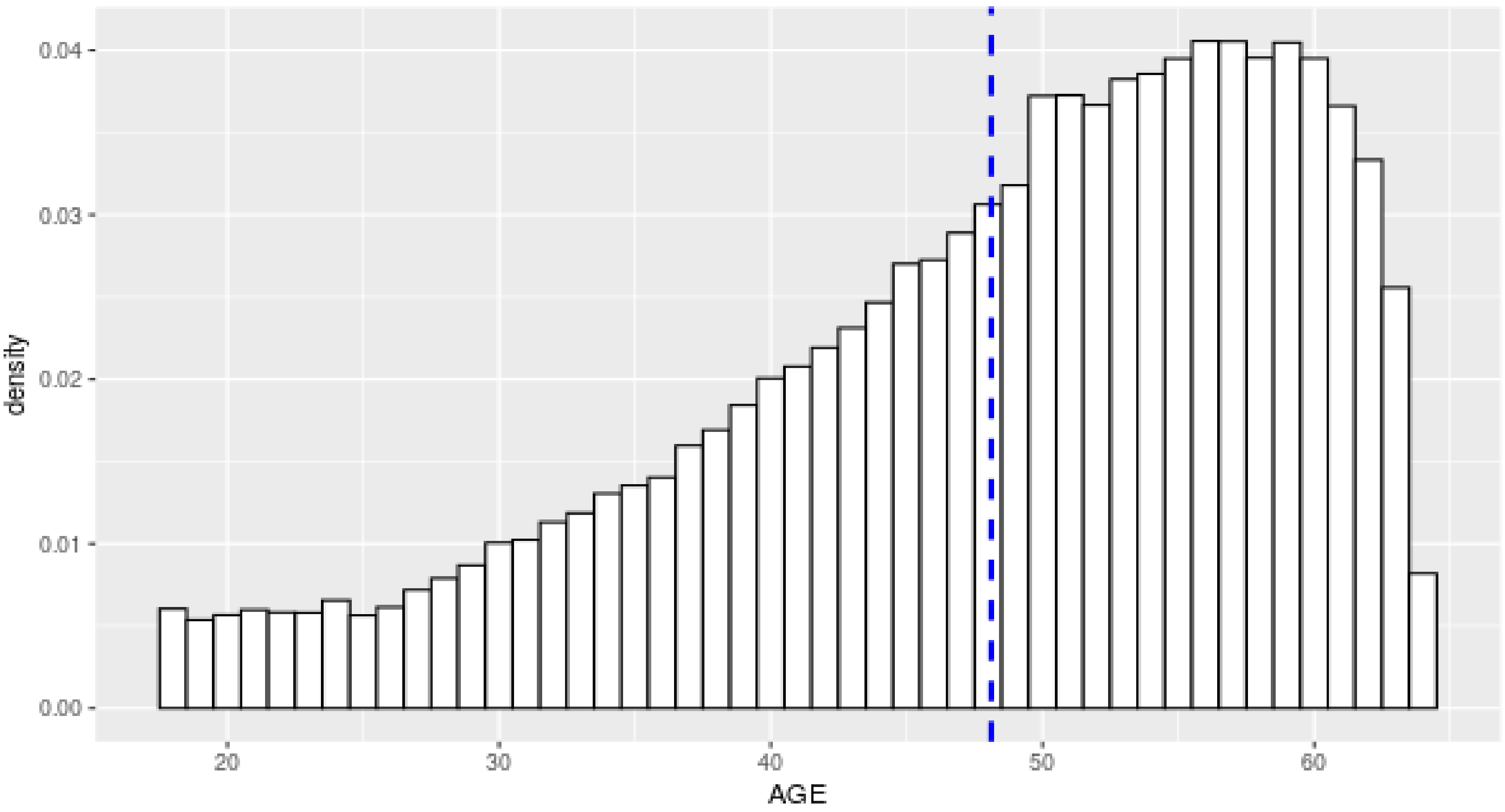

Figure S2: Age distribution of the cohort, with mean age 48 and median age 50. Notably, the Commercial Claims and Encounters database does not include any individuals ≥65 years of age.
